# The Neuronal Correlates of Indeterminate Sentence Comprehension: An fMRI Study

**DOI:** 10.3389/fnhum.2016.00614

**Published:** 2016-12-20

**Authors:** Roberto G. de Almeida, Levi Riven, Christina Manouilidou, Ovidiu Lungu, Veena D. Dwivedi, Gonia Jarema, Brendan Gillon

**Affiliations:** ^1^Department of Psychology, Concordia UniversityMontreal, QC, Canada; ^2^Department of Comparative and General Linguistics, University of LjubljanaLjubljana, Slovenia; ^3^Unité de Neuroimagerie Fonctionnelle, Institute Universitaire de Gériatrie de Montréal, Université de MontréalMontreal, QC, Canada; ^4^Department of Applied Linguistics, Brock UniversitySt. Catharines, ON, Canada; ^5^Centre de Recherche, Institut Universitaire de Gériatrie de Montréal, Université de MontréalMontreal, QC, Canada; ^6^Department of Linguistics, McGill UniversityMontreal, QC, Canada

**Keywords:** indeterminate sentences, semantic coercion, compositionality, pragmatics, fMRI, inferior frontal gyrus, superior temporal gyrus, anterior cingulate cortex

## Abstract

Sentences such as *The author started the book* are indeterminate because they do not make explicit what the subject (*the author*) started doing with the object (*the book*). In principle, indeterminate sentences allow for an infinite number of interpretations. One theory, however, assumes that these sentences are resolved by *semantic* *coercion*, a linguistic process that forces the noun *book* to be interpreted as an *activity* (e.g., *writing the book*) or by a process that interpolates this activity information in the resulting enriched semantic composition. An alternative theory, *pragmatic*, assumes classical semantic composition, whereby meaning arises from the denotation of words and how they are combined syntactically, with enrichment obtained via pragmatic inferences beyond linguistic-semantic processes. Cognitive neuroscience studies investigating the neuroanatomical and functional correlates of indeterminate sentences have shown activations either at the ventromedial pre-frontal cortex (vmPFC) or at the left inferior frontal gyrus (L-IFG). These studies have supported the semantic coercion theory assuming that one of these regions is where enriched semantic composition takes place. Employing functional magnetic resonance imaging (fMRI), we found that indeterminate sentences activate bilaterally the superior temporal gyrus (STG), the right inferior frontal gyrus (R-IFG), and the anterior cingulate cortex (ACC), more so than control sentences (*The author wrote the book*). Activation of indeterminate sentences exceeded that of anomalous sentences (…*drank the book*) and engaged more left- and right-hemisphere areas than other sentence types. We suggest that the widespread activations for indeterminate sentences represent the deployment of pragmatic-inferential processes, which seek to enrich sentence content without necessarily resorting to semantic coercion.

## Introduction

A hallmark of linguistic communication is that sentences are often indeterminate: their *intended* messages cannot be obtained by simply resorting to what they explicitly *say*, that is, to the meaning of their constituent words and how those meanings combine. For example, a sentence such as (1a) can be taken to express the idea that John wants *to drink* or *to buy* a beer, just like (1b) can be taken to express that Mary began *reading* or *writing* the book.

(1) a. John wants a beer;
b. Mary began the book.

In fact, there are many possible interpretations for sentences (1a) and (1b) beyond what they say. What is not clear is the source of information that is supposed to enrich the content of these sentences beyond what their constituent words and their modes of combination provide. More specifically, how does *Mary began the book* come to be understood as something like *Mary began to read the book*? And, more pertinent to our present goals, what are the neurological resources deployed in the comprehension of these sentences?

Thus far, there have been two prevalent theoretical views on how indeterminate sentences such as (1a) and (1b) are understood. The *coercion* theory (e.g., Pustejovsky, 1995; Jackendoff, 1997) assumes that semantic information represented within the complement noun *book* (e.g., that books are read) contributes content to the sentence and, thus, licenses an *enriched* semantic representation, such as *Mary began reading the book*. A related view assumes that a semantic operation—*type shifting* or *type coercion*—is responsible for changing the reading of the object noun from that of an *entity* (*book*) into that of an *activity* (thus denoting something done with the book—such as *reading the book*)^1^. An alternative to both versions of the coercion theory is one which we call *pragmatic* (de Almeida and Dwivedi, 2008; de Almeida and Riven, 2012; see also Fodor and Lepore, 2002). This theory assumes that the way in which a sentence such as (1b) is understood arises from the application of general pragmatic principles, where the constituents and the structure of the sentence are initially taken at face value (see also Dölling, 2014).

With regards to the neurological correlates of indeterminate sentence comprehension, different studies point to the engagement of different neuroanatomical regions as the main source of indeterminacy resolution. For instance, magnetoencephalography (MEG) studies suggest that the ventromedial prefrontal cortex (vmPFC) is the main site of indeterminacy resolution—in fact, the site of semantic coercion (Pylkkänen and McElree, 2007; Pylkkänen et al., 2009). The only study employing functional magnetic resonance imaging (fMRI; Husband et al., 2011), however, found no evidence for the engagement of the vmPFC, suggesting instead that the left inferior frontal gyrus (L-IFG; BA 45) is the site of coercion processes. The present study also employed fMRI to further investigate the neurological correlates of indeterminacy resolution. What differentiates our study from previous ones is that we were also interested in understanding whether indeterminate sentences are resolved by coercion or whether they involve pragmatic processes. To this end, we employed a larger number of experimental control conditions and a whole-brain analysis in order to obtain a broader picture of the neuroanatomical map of indeterminate sentences, in contrast to other sentence types.

While our main goal is to establish the neurological resources deployed in the resolution of indeterminate sentences, our empirical investigation also aims to contribute to a better understanding of the theoretical underpinnings of indeterminacy—whether sentences are resolved by semantic coercion or pragmatic processes. We thus begin by discussing what is at stake in the investigation of indeterminate sentence comprehension: how the brain might compose meanings of sentences.

### Two Views on Semantic Composition

The process of understanding a sentence requires at a minimum that the meaning of its constituent words or morphemes be put together according to its structure—what is known as *compositionality* or *semantic composition* (for a review see e.g., Partee, 1984; Fodor and Lepore, 2002; Szabó, 2012). The two views on indeterminate sentence resolution briefly presented above differ mainly with regards to what they take to be the nature of semantic composition processes.

The coercion theory by and large adopts a view known as *enriched compositionality* (Pustejovsky, 1995; Jackendoff, 1997). With regards to sentences such as those in (1), the assumption is that indeterminacy arises from a *mismatch* between the requirements of the verb and the semantic nature of its internal argument. A verb such as *begin*, for instance, arguably requires an activity complement such as a gerundial phrase (*reading the book*), an infinitival phrase (*to read the book*), or even a noun complement denoting an activity (e.g., *the lecture*). But when only a noun phrase (NP) complement denoting an entity (*the book*) appears, the mismatch is assumed to be resolved by *coercion* (Pustejovsky, 1995). Thus, although activities such as *drinking* or *reading* are not specified overtly in (1a) and (1b), forms such as *want x* or *begin x* can be taken to denote something like *want/begin to do with x what is typically done with x*, with interpolation of a default activity into the resulting composition. Alternatively, the process of *coercion* relies simply on type-shifting; that is, modifying the interpretation of the *entity* (e.g., *book*) into that of an *activity* in order to conform with the verb requirements (akin to Partee, 1986). Although there are numerous variations on these approaches to coercion processes (e.g., Traxler et al., 2005; Pylkkänen, 2008)—and in particular whether or not *interpolation* occurs—what is relevant for the present purposes is the postulation that the resolution of indeterminacy is driven primarily by linguistic-internal mechanisms. And common to all theories of coercion is the proposal that the operation—triggered by a mismatch between verb and complement—yields an enriched composition.

In contrast, the pragmatic-inferential theory assumes that the initial representation of a sentence is only a function of the meaning of its constituent words and how they are structured together—in syntax or logical form—a position commonly referred to as *classical compositionality* (Fodor and Lepore, 2002). According to this view, enriched interpretations of sentences such as (1b) are possible only because the linguistic form leaves informational “gaps” which serve as triggers for pragmatic inferences (Fodor and Lepore, 2002; de Almeida and Dwivedi, 2008; de Almeida and Lepore, in press). Although this view does not make particular claims about how pragmatic inferences are carried out (for alternatives see e.g., Carston, 2002; Hobbs, 2004), the key idea is that any conceptual enrichment that might occur as a function of semantic indeterminacy is resolved by appealing not to a coercion operation, but to pragmatics (see also Dölling, 2014). Essentially, pragmatic inferences are not taken to be *constitutive* of the meaning of the sentence but a natural consequence of it: they are inferences one draws upon hearing a sentence that leaves information to be desired or implicated (see Grice, 1989).

In summary, while what we called coercion theory takes type-shifting and interpolation to be key elements of enrichment—with varying degrees of contribution from other knowledge systems (see e.g., Traxler et al., 2005)—what we called the pragmatic theory is committed to classical compositionality at the linguistic level and defers enrichment to pragmatic inferences on the assumption that the linguistic system is uninformed about possible enriching events.

### Empirical Studies

There have been numerous behavioral studies investigating how indeterminate sentences might be processed. These have involved experimental paradigms such as self-paced reading (McElree et al., 2001; de Almeida, 2004; Leitão et al., 2010) and eye-tracking (Traxler et al., 2002, 2005; Pickering et al., 2005; McElree et al., 2006; Katsika et al., 2012). The majority of these studies have demonstrated that there are “costs” associated with processing indeterminate sentences such as (2a) compared to preferred sentences (2b) at the noun (*memo*) and for post-noun positions (e.g., *before*). These results have been taken as evidence of semantic coercion assuming that the extra time required to read the indeterminate sentence is due to the process of interpolation of semantic information, yielding an interpretation such as (2d).

(2) a. The secretary began the memo before the annual sales conference (indeterminate);
b. The secretary typed the memo before the annual sales conference (preferred);
c. The secretary read the memo before the annual sales conference (non-preferred);
d. The secretary began [*typing*] the memo before the annual sales conference.

While these studies have been useful in calling attention to the phenomenon, their results have been conflicting (see e.g., de Almeida, 2004; Pickering et al., 2005) and they are compatible with alternative explanations (e.g., de Almeida and Dwivedi, 2008).

Perhaps more important for our current purposes are studies employing cognitive neuroscience methods, which can complement behavioral studies while also potentially helping us dissociate the very source of behavioral differences. Most relevant to the present study were the studies by Pylkkänen and McElree (2007), using MEG, and by Husband et al. (2011), using fMRI, both of which aimed to identify the neural networks implicated in the resolution of indeterminacy, assuming that it necessarily involves coercion^2^.

In their MEG study, Pylkkänen and McElree (2007) compared indeterminate sentences such as (3a) to anomalous sentences such as (3b) and preferred controls such as (3c) in a task that required participants to judge the acceptability of these sentences.

(3) a. The journalist began the article after his coffee break (*indeterminate*);
b. The journalist astonished the article after his coffee break (*anomalous*);
c. The journalist wrote the article after his coffee break (*preferred*).

They found that, relative to the other conditions, indeterminate sentences produced a unique response in what they called the anterior midline field (AMF), hypothesized to be at the vmPFC. This response occurred at 350–500 ms after the presentation of the NP *the article*. The effect was flanked by bilateral temporal activation, with an outgoing field to the right temporal region and an entering field from the left temporal region. Given this differential activation of indeterminate sentences, the authors suggested that the AMF, particularly the vmPFC, is the locus of enriched composition.

Most recently, Husband et al. (2011) conducted an fMRI study with sentences modified from Pylkkänen and McElree (2007), with the addition of a syntactically anomalous condition such as *The novelist write the book before the break*. Similar to Pylkkänen and McElree (2007) method, participants were asked to judge the acceptability of sentences during scanning. They found differential activations between indeterminate and control sentences in three main areas. In two of these areas—left middle-temporal gyrus (L-MTG) and left inferior parietal lobe (L-IPL)—they found deactivations. Their main finding was that indeterminate sentences significantly activated the L-IFG (BA 45) relative to controls, leading the authors to conclude that this region represents the locus of coercion. No differential activation was found in the vmPFC, contra Pylkkänen and McElree’s (2007) AMF result.

Husband et al. (2011) explained their failure to replicate Pylkkänen and McElree’s (2007) findings in terms of technical disparities. Whereas MEG is sensitive to the temporal components of processing, fMRI is better suited to localization mapping. In addition, the vmPFC activations measured by fMRI are prone to susceptibility artifacts (Ojemann et al., 1997), which may have attenuated the contrast between indeterminate and control sentences. Thus, each study has provided unique insights concerning the neural substrates of indeterminate sentence processing. The MEG results suggest that interpretation engages left-temporal, right-temporal and medial-frontal areas, and that the time course of processing progresses from left to right to medial regions (Pylkkänen and McElree, 2007). The fMRI data suggest that the L-IFG emerges as a critical region of processing, rather than the vmPFC.

Although we regard these data as informative, the neurological underpinnings and, more importantly, the source of indeterminacy resolution remain unresolved. One possible explanation for the difference between the MEG and fMRI results is that the proposed AMF activation represents core processes occurring in medial frontal regions other than the vmPFC. If this is indeed the case, additional fMRI data are needed to explore these regions in greater detail. In addition, although Pylkkänen and McElree (2007) describe significant processes occurring in right temporal areas, the role of the right hemisphere (RH) during indeterminate sentence processing has yet to be further investigated. The only fMRI study on indeterminate sentence processing to date (Husband et al., 2011) has found effects primarily in the L-IFG, and has restricted the region of interest (ROI) analyses to the vmPFC and traditional language regions in the left hemisphere (LH; e.g., L-IFG). Moreover, neither of the neuroscience studies we discussed above was designed specifically to contrast *coercion* with *pragmatic* accounts of indeterminacy resolution: rather they aimed to find the sources of *coercion*, under the assumption that any neural activation differences between indeterminate sentences and controls would constitute evidence for coercion. Yet indeterminate sentences differ from control, fully determinate sentences in many respects, including the syntactic structure of their verb phrases (see de Almeida and Dwivedi, 2008) which might account for their processing differences. Moreover, an exploration of other brain regions—beyond the vmPFC and L-IFG—during indeterminate sentence comprehension is important to investigate alternative hypotheses to coercion, in particular the hypothesis that indeterminate sentences are resolved pragmatically.

### The Present Study

The primary goal of the present fMRI study was to determine the brain areas recruited in attempting to resolve indeterminate sentences. We reasoned that the neuroanatomical signature of indeterminate sentences would be fundamentally different from that of determinate sentences on the assumption that indeterminate sentences might trigger the search for implicatures akin to what is triggered when sentences flout a conversational maxim (Grice, 1989)—when, for instance, sentences are less informative than they need to be or are purposefully ambiguous. Thus, our first goal was to obtain an activation map for indeterminate sentences and various controls—ranging from fully determinate sentences to syntactic and semantic anomalies. We also aimed to examine the involvement of medial and right-hemispheric brain regions, which have been linked to different types of pragmatic processes, in addition to examining the involvement of traditional language areas. We regard this broader approach as essential for understanding the effects found in the majority of behavioral studies, with important implications for the functional and neurological bases of sentence comprehension more generally. In addition, we expected that such data would complement the time-course and localization effects observed with MEG (Pylkkänen and McElree, 2007), thus providing converging evidence on the nature of indeterminate sentence processing.

Although our main hypotheses are not tied to specific neuroanatomical regions—for we aim to find differences in the neuronal correlates between indeterminate and other types of sentences—we will discuss rather briefly the literature on the neuroanatomy of language processing and how it helps us lay out our hypotheses. Numerous reviews and meta-analytic studies have shown that large neuroanatomical regions beyond the classical linguistic areas (e.g., Broca’s and Wernicke’s) are involved in linguistic processes serving comprehension and production (e.g., Bookheimer, 2002; Friederici, 2002, 2011; Gernsbacher and Kaschak, 2003; Indefrey and Cutler, 2004; Stowe et al., 2005; Hickock and Poeppel, 2007; Meyer, 2009; Binder et al., 2011). Several studies manipulating sentence complexity emphasize the role of the L-IFG (BA 44/45) in syntactic structuring and syntactic memory (e.g., Stromswold et al., 1996; Kang et al., 1999; Ni et al., 2000; Friederici et al., 2003; Fiebach et al., 2005; Makuuchi et al., 2009; Friederici, 2011). Imaging studies on semantic aspects of word and sentence comprehension have also pointed to the dominant role of the LH, in particular the superior temporal gyrus (STG) and the MTG (e.g., Binder et al., 1997, 2009; Bookheimer, 2002; Friederici et al., 2003; Damasio et al., 2004; Berwick et al., 2013). Sub-regions in booth L-IFG and STG/MTG and their interconnecting dorsal and ventral pathways are seen as constituting a default language network for the vast majority of right-handed speakers (for review see Friederici, 2011).

While linguistic processes involved in language comprehension appear to rely on the LH default language network, there are several issues with how these processes map onto higher mechanisms of language interpretation. For instance, the neuroanatomical resources involved in pragmatic processes are less than clear. Studies with RH-damaged patients have shown diminished comprehension of implicit requests, irony, and metaphors (e.g., Champagne-Lavau and Joanette, 2009). Although many RH-damaged patients show generally spared syntactic and lexical-semantic abilities (e.g., Dronkers et al., 2004), they also show a marked difficulty with implicit statements violating the maxim of quantity (*make your contribution as informative as required for the purposes of the exchange*; Grice, 1989) compared to statements that make explicit assertions (Champagne et al., 2003). Moreover, RH-damaged patients have difficulty drawing inferences from connected sentences while having preserved ability to understand individual sentences (Brownell et al., 1986; for review see also Joanette et al., 2008). These results from patient studies are compatible with several neuroimaging experiments investigating discourse comprehension, which have found activation in large RH and frontal areas (for review see Gernsbacher and Kaschak, 2003). It is important to point out that discourse comprehension processes require the integration of diverse sources of information beyond sentence meaning and thus might engage areas that underlie inferential processing. For instance, studies have shown significant RH activation when subjects have to make inferences about implied (vs. explicit) events (Virtue et al., 2006), when they have to generate “unusual relationships” between verbs and nouns (e.g., producing *throw* for the noun *dish*; Seger et al., 2000), and when they have to interpret stories (Binder et al., 2011).

While hemispheric asymmetry in language processing is clear, many have questioned the so-called “right-hemisphere hypothesis”, i.e., the hypothesis that the RH is primarily involved in high-level, pragmatic processes (for a review see e.g., Stemmer, 2016). A version of this hypothesis, however, calls not for *greater* involvement of RH compared to LH, but for *different roles* for both hemispheres—with the LH more involved in fine-grained, literal interpretations, and the RH involved in more “coarse” and figurative interpretations (Jung-Beeman, 2005). Consistent with this hypothesis, Schmidt and Seger (2009) found that the right insula and the right inferior frontal gyrus (R-IFG) are significantly activated for metaphors compared to literal sentences. Similar findings were obtained by Bambini et al. (2011)—with overall greater bilateral activation of IFG, the insula, and the anterior cingulate cortex (ACC) for metaphors compared to literal expressions. Bambini et al. (2011) also found greater involvement of right superior temporal sulcus (STS) and middle FG in metaphor processing, suggesting that quintessential pragmatic processes engage different RH neural substrates—or engage them to a larger extent—when compared to literal language (see also Bottini et al., 1994; Kacinik and Chiarello, 2007)^3^. We see a similar pattern even in studies showing LH-dominance in the comprehension of metonymy (Rapp et al., 2011): the R-IFG shows significant activation for metonymic (*Hitchcock is worth watching*) expressions beyond what is required to activate literal expressions (*Hitchcock is dead*). Other forms of figurative language processing—such as irony and sarcasm—also suggest the activation of bilateral networks, with significant RH clusters compared to literal controls (for a review see Bohrn et al., 2012).

Results from these studies with figurative language, which arguably require inferences or the search for what is implicated, are compatible with those that employed semantically or pragmatically odd sentences, which also involve reinterpretation or the detection of a mismatch in semantic composition (e.g., Ni et al., 2000; Friederici et al., 2003). These latter studies show strong bilateral activation of both STG and insula, suggesting that processes beyond simple LH-driven semantic composition might be recruited for the interpretation of anomalous sentences. In fact, it has been suggested that semantic composition proper (what we refer to as *classical compositionality*) involves left temporal structures, in particular the anterior temporal lobe (ATL; see Vandenberghe et al., 2002; Hickock and Poeppel, 2007; Lau et al., 2008). Collectively, these studies suggest that LH and RH structures are involved in pragmatic enrichment, above and beyond what is required to compose canonical sentences.

The picture that emerges from the literature on neurological implementation of linguistic processes allows us to hypothesize that regions engaged in pragmatic and high-level cognitive processes would be more involved in the comprehension of indeterminate sentences (e.g., *The author started the book*) above and beyond their engagement in the comprehension of preferred sentences (e.g., *The author wrote the book*). More specifically, we predicted that, if pragmatic processes are called for to enrich indeterminate sentences, this should engender greater activation of bilateral temporal and frontal areas (compatible with the vmPFC/AMF effects found by Pylkkänen and McElree, 2007), beyond the engagement of these areas in preferred sentences. Conversely, if the resolution of indeterminacy relies primarily on semantic coercion, we should not expect to see comparatively greater involvement of bilateral temporal and frontal regions during online comprehension. We should rather expect greater involvement of the left anterior temporal cortex (AT)—the STG, and the MTG—traditionally associated with lexical representations and possibly semantic composition (Vandenberghe et al., 2002; Lau et al., 2008). Moreover, what the study by Husband et al. (2011) has suggested is that the L-IFG should play a significant role in indeterminacy resolution for it is at this site where the alleged mismatch detection and repair take place.

We should note that these hypotheses can only be laid down in broad strokes, for we cannot distinguish between sources of indeterminacy resolution without a clear understanding of what types of *computations* the neuronal circuits perform. Moreover, there are numerous potential neurological sources for semantic composition—at least the vmPFC, the L-IFG, and the AT—and it is possible that these areas make different contributions to the process. But it is also possible that the relative involvement of all these areas might signal fundamentally different types of compositional processes—say, processes ranging from lexical retrieval to structure building and interpretation. In the present study, we restrict our hypothesis to investigating specifically the composition (and possible enrichment) of indeterminate sentences. With these caveats in mind, we take the neuronal signature of the indeterminate sentence resolution to suggest what types of information are recruited; currently, the alternatives are: (a) purely linguistic, coercive operations restricted to the vmPFC or the L-IFG; and (b) pragmatic-inferential processes of enrichment, with the engagement of diverse brain areas bilaterally.

In addition to examining the networks activated by indeterminate sentences relative to preferred controls, we sought to inform the pragmatic hypothesis by observing how indeterminate sentences pattern relative to anomalous sentences, such as *The author drank the book*. Such sentences violate beliefs about real-world situations, and therefore impose special demands on pragmatic processes—i.e, possibly by resorting to a figurative interpretation^4^. Accordingly, we predicted that insofar as indeterminate sentences impose analogous demands for pragmatic processes, they should pattern together with such anomalous sentences, particularly in the RH. This hypothesis is based on similar cases of violations, such as *The woman ironed a kiss*, which engage the RH significantly more than controls (Kuperberg et al., 2000). Finally, we employed three additional conditions: (1) non-preferred sentences such as *The author read the book*; (2) full-VP sentences that included the indeterminate verb and the preferred verb, as in *The author started writing the book*; and (3) syntactically anomalous sentences, such as *The author yawned the book*. Each of these conditions contrasts with indeterminate sentences in unique ways, allowing us to explore several secondary hypotheses. In the following sections, we describe each of these sentences in more detail and outline how they are involved in our analyses.

## Materials and Methods

### Norming Study

Our norming study was framed by three main goals. First, we wanted to gather acceptability rating data for the 648 sentences employed in the fMRI experiment to ensure that our canonical sentences would be rated within the normal range in contrast with the anomalous conditions. Our second goal was to obtain RT data for each sentence, segment by segment, to allow us to contrast our materials with those employed in the psycholinguistics literature. And our third goal was to obtain a behavioral complement to our fMRI experiment. The latter was designed to elicit natural language processing without the complication of a secondary task, be it psychomotor (e.g., button-pressing in self-paced reading) or cognitive (grammaticality ratings). Sentence rating tasks in particular may be problematic because they require participants to engage in a metalinguistic mode of processing, thus confounding the signal of online comprehension processes. To date, both the MEG (Pylkkänen and McElree, 2007) and fMRI (Husband et al., 2011) studies of indeterminate sentences have employed grammaticality judgment tasks, leaving open the possibility that the activation that indeterminate sentences elicited may be related to metalinguistic processing. We therefore chose to obtain behavioral data (ratings, RTs) separately in order to diminish the impact of offline judgments on our fMRI data.

#### Materials

The experimental sentences were created by first asking a group of 60 students to fill in the blanks of frames such as *The* ___ *started the*___ (for the indeterminate condition) and *The*___*started*___*ing the*____ (for the preferred and full-VP conditions). We then selected the most frequently used agent/main-verb/object combination to construct a stimulus set consisting of 108 sentence frames (e.g., *The author…the book*) that differed across six verb conditions (see Table 1): (1) Preferred; (2) Non-preferred; (3) Full-VP; (4) Indeterminate; (5) Syntactically anomalous; and (6) Pragmatically anomalous. Each of these conditions provided unique opportunities to investigate the nature of indeterminate sentence processing. The first three conditions represented canonical alternatives to the indeterminate sentence. The preferred sentences included verbs that were judged (see below) to best fit with an entity NP complement (*wrote*); the non-preferred sentences included verbs that were judged plausible but less preferred (*read*); and the full-VP condition included both the indeterminate verb and an activity complement (*started writing*). The last two conditions provided anomalous contrasts for indeterminate sentences, one introducing a syntactic/semantic violation, namely, an intransitive verb (*yawned*), and the other introducing a semantic/pragmatic violation (*drank*), both followed by the same complement employed in the other conditions (*the book*). The 108 sentence frames thus appeared in six conditions, making a total of 648 sentence tokens. These were divided into six lists, with 18 tokens of each condition in each list. In addition, a set of 62 filler sentences was added to each list, for a total of 170 sentences per list.

**Table 1:** **Acceptability data (by items) for sentences used in the fMRI experiment**.

Sentence type	Sample	*df*	*M*	*SD*	*SD pooled^a^*	*d^a^*
Preferred	The author wrote the book	107	4.76	0.33	–	–
Full-VP	The author started writing the book	107	4.54	0.46	0.63	0.35
Non-preferred	The author read the book	107	4.44	0.59	0.68	0.48
Indeterminate	The author started the book	107	3.54	1.06	0.84	1.46
Syntactically anomalous	The author yawned the book	107	1.23	0.27	0.55	6.42
Pragmatically anomalous	The author drank the book	107	1.46	0.49	0.64	5.15

*^a^The *preferred* sentence type was used to calculate *SD* pooled and *d* values for the subsequent conditions*.

#### Ratings

A group of 89 students who did not participate in the other tasks was asked to judge the acceptability of the sentences on a 5-point scale. They all gave written informed consent and participated for course credit as part of the Concordia Psychology Participant Pool. Table 1 includes the descriptive statistics for the six sentence conditions by item. Results from a series of pairwise *t*-tests indicate that compared to preferred sentences, all other non-anomalous conditions were judged less acceptable (Indeterminate: *M*_D1_ = −1.23, *t1*_(88)_ = −24.72, *p* < 0.004^5^; *M*_D2_ = −1.22, *t2*_(107)_ = −11.33, *p* < 0.004; Non-preferred: *M*_D1_ = −0.32, *t1*_(88)_ = −8.11, *p* < 0.004; *M*_D2_ = −0.33, *t2*_(107)_ = −5.54, *p* < 0.004; and full-VP: *M*_D1_ = −0.22, *t1*_(88)_ = −7.18, *p* < 0.004; *M*_D2_ = −0.22, *t2*_(107)_ = −5.03, *p* < 0.004).

In order to evaluate the degree to which each condition departed from the canonical preferred condition, we calculated mean-difference effect sizes (*d*). Although indeterminate sentences deviate from preferred sentences (*d* = 1.46), this difference did not reach the magnitude observed in the two anomalous conditions (*d* > 5.15). As can be seen in Table 1, even non-preferred and full-VP sentences deviate from the preferred condition, although none of these conditions constitute syntactic or semantic violations. These data suggest that, although indeterminate sentences are judged less canonical than preferred and non-preferred sentences, similar to other studies (e.g., Husband et al., 2011), they are within the range of acceptable sentences.

#### Reading Time

Another group of 83 participants, all of whom gave informed consent, completed a word-by-word self-paced reading task. The six sentence conditions and a set of 65 filler sentences were presented in random order. Among the fillers, 25 sentences were followed by Yes/No questions to ensure that the participants remained attentive throughout the task.

Reading time data for all six conditions are summarized in Table 2. Note that raw data were normalized by removing outliers with values corresponding to ±2 standard deviations from the mean of each condition (3.5% below and 7.1% above). We conducted a set of one-way ANOVAs and pairwise contrasts, comparing preferred, non-preferred and indeterminate sentences, at the verb, determiner and noun positions. We restricted our principal analysis to these three conditions in order to ease comparison with psycholinguistic studies reviewed above. Given these multiple comparisons, we adjusted our threshold of significance for each contrast to 0.004.

**Table 2:** **Mean reading times (and standard deviations) for the six sentence types obtained in the norming task**.

Verb type	Sentence position		
	Verb	Determiner *the*	Noun *book*
Preferred (*wrote*)	355 (86)	333 (66)	454 (186)
Non-preferred (*read*)	359 (87)	336 (70)	470 (192)
Indeterminate (*started*)	365 (95)	342 (68)	480 (192)
Full VP (*started writing*)	377 (95)	348 (65)	476 (227)
Syntactically anomalous (*yawned*)	360 (96)	365 (99)	515 (229)
Pragmatically anomalous (*drank*)	365 (99)	347 (68)	492 (227)

##### Verb

At the verb position, there was no difference between sentences in the participants analysis, *F1*_(5,410)_ = 1.05, *p* = 0.22, *MSE* = 1821.43, although in the items analysis there was a significant effect, *F2*_(5,535)_ = 2.41, *p* = 0.045, *MSE* = 2744.80. The sentence condition accounted for only 2% of the variation in verb reading times, thus, we do not consider the effect to be substantively significant.

##### Determiner

At the determiner position, both participants and items analyses revealed statistically significant main effects, *F1*_(5,410)_ = 10.42, *p* < 0.001, *MSE* = 1862.24, partial-$\eta_{1}^{2}$ = 0.11; *F2*_(5,535)_ = 6.83, *p* < 0.001, *MSE* = 1783.45, partial-$\eta_{2}^{2}$ = 0.06. Pairwise comparisons revealed that reading times for the indeterminate condition were significantly longer than for the preferred condition (*M*_D_ = 9.40 ms) at the conventional threshold in the participants analysis, *t1*_(82)_ = 2.70, *p* = 0.008, but not in the items analysis, *t2*_(107)_ = 1.88, *p* = 0.063.

##### Noun

There was also a main effect of sentence at the noun position, *F1*_(5,410)_ = 4.31, *p* < 0.01, MSE = 13884.62, partial-$\eta_{1}^{2}$ = 0.05; *F2*_(5,535)_ = 3.56, *p* < 0.05, MSE = 43823.33, partial-$\eta_{2}^{2}$ = 0.03. Pairwise comparisons showed that, while reading times for the indeterminate sentence exceeded that of the preferred sentence (*M*_D_ = 25.61 ms), this difference did not surpass the adjusted threshold of significance. Nevertheless, the comparison was significant at the conventional alpha level for the items analysis, *t2*_(107)_ = 2.64, *p* = 0.009, but not the participants analysis, *t1*_(82)_ = 1.73, *p* = 0.087.

These RT data are largely in keeping with effects observed in the psycholinguistics literature, which typically demonstrates that indeterminate sentences take longer to process post-verbally than preferred sentences (e.g., McElree et al., 2001), although not consistently so (de Almeida, 2004; Pickering et al., 2005; Leitão et al., 2010).

In addition to replicating the RT effects found in other behavioral studies, we conducted additional analyses to further characterize our materials and guide hypotheses. Table 3 summarizes the results of dependent-samples *t*-tests comparing reading times of indeterminate sentences to all other conditions at the noun region (e.g., *book*)—i.e., the initial locus of indeterminate processing difficulty. These comparisons show that, with the exception of the preferred contrast, indeterminate sentences are not statistically costly relative to any other condition, including non-preferred and full-VP sentences. Moreover, indeterminate sentences were found to be processed more easily than both anomalous conditions, but only the contrast with syntactic anomalies achieved statistical significance.

**Table 3:** **Pairwise analyses at the noun region (e.g., *book*), subtracting indeterminate sentences from all other conditions in the self-paced reading norming task**.

Sentence type	*M_D_**(ms)***	By participants	By items
Preferred	−25.61	*t*_(82)_ = −1.73, *p* = 0.087	*t*_(107)_ = −2.64, *p* = 0.009
Non-preferred	−10.16	*t*_(82)_ = −1.20, *p* = 0.233	*t*_(107)_ = −0.46, *p* = 0.649
Full-VP	−4.27	*t*_(82)_ = −0.30, *p* = 0.765	*t*_(107)_ = −0.64, *p* = 0.525
Syntactically anomalous	34.87	*t*_(82)_ = 2.76, *p* = 0.007	*t*_(107)_ = 3.09, *p* = 0.003
Pragmatically anomalous	12.01	*t*_(82)_ = 1.23, *p* = 0.222	*t*_(107)_ = 1.58, *p* = 0.117

These results converge with our ratings data, which showed a continuum of acceptability judgments from preferred sentences (most felicitous) to syntactically anomalous sentences (least felicitous), and that indeterminate sentences lie closer to the felicitous end of this spectrum. Having collected both off-line and on-line response measures for our materials, we set out to conduct an fMRI experiment requiring participants to passively read the stimuli, thus allowing us to track language comprehension processes without the potential contaminations of an extraneous task.

### The fMRI Experiment

As we have described above, our principal hypotheses concern patterns of activation for indeterminate sentences compared with preferred sentences and pragmatic violations. But we have also included several conditions that would allow us to explore secondary hypotheses regarding the nature of indeterminate sentence processing. We now elaborate on our main hypotheses and the contrasts with each condition.

#### Hypotheses

##### Indeterminate sentences

We have designed the present experiment aiming to understand the pattern of activation for indeterminate sentences and thus gain insights into the nature of the neurocognitive resources employed in the resolution of indeterminacy. Given the inconsistent neuroanatomical correlates found in the two studies employing fMRI and MEG, we sought out first to establish the map of indeterminacy resolution. In addition we aimed to gather support for either the coercion or the pragmatic theory. Support for the coercion theory would be rather restricted to greater linguistic operations either at the L-IFG (Husband et al., 2011) or at the vmPFC (Pylkkänen and McElree, 2007). We predicted that activations beyond these regions, and fundamentally in contrast with other sentence conditions, could be taken for operations that were not restricted to linguistic-semantic processing, but also involving pragmatic processes of indeterminacy resolution. We predicted in particular activations to involve temporal and frontal regions bilaterally, which would be more consistent with patterns of activation obtained in studies involving pragmatic processes of non-literal sentence interpretation and discourse processing. Crucial to our hypotheses are also the contrasts with the following other five conditions.

##### Preferred

Preferred sentences represent the canonical form. They are employed as the standard control condition for indeterminate sentences, and have been shown to be processed more easily than indeterminate sentences (e.g., McElree et al., 2001; Traxler et al., 2002)—a result which we have replicated above with both offline and online response measures. Consistent with our pragmatic-inferential hypotheses, we predicted that the indeterminate and preferred sentences would differ especially in terms of pragmatic resources recruited during sentence processing, with the former eliciting comparatively greater activation in fronto-temporal areas bilaterally.

##### Pragmatically anomalous

Our acceptability judgments showed that what we called pragmatically anomalous sentences are regarded as infelicitous compared to preferred and indeterminate sentences. However, although they engendered numerically longer RTs relative to indeterminate sentences (*M*_D_ = 12 ms), this difference was not statistically significant. We hypothesized that both sentences require pragmatic inferences to build an acceptable interpretation, and therefore, indeterminate sentences should pattern together with pragmatically anomalous sentences.

##### Non-preferred

Although syntactically well formed, non-preferred sentences describe activities that are less strongly associated with the agent in the sentence, and are therefore rated lower than preferred sentences. Although they are grammatical, their interpretation requires accessing non-dominant meanings, which makes them more demanding than preferred sentences (de Almeida, 2004). Therefore, it would be expected for non-preferred sentences to pattern primarily with preferred sentences but with attenuated differentiation with indeterminate sentences, as was demonstrated with our norming data.

##### Full-VP

Full-VP sentences are both syntactically well formed and pragmatically felicitous. However, our RT data show that they take longer to process than preferred sentences at the object position and that they are not significantly faster than indeterminate sentences. Full-VP sentences are structurally and semantically more complex then preferred sentences, and thus might require additional computations. These factors may have increased processing times, even though the sentences were judged to be felicitous. Accordingly, we predicted that full-VP sentences would recruit more linguistic resources than preferred sentences as a function of their complexity—in particular due to the need to compose the more complex VP. They should therefore diverge from indeterminate sentences pragmatically: rather than leaving the nature of the event to be inferred, they make it explicit.

##### Syntactically anomalous

These sentences were formed by using non-alternating intransitive verbs with complements. These sentences were judged to be the least felicitous and have generated the longest processing latencies. There are at least two possibilities for how the parser might deal with such structural violations: (1) pursue effortful repair processes; or (2) reject the sentence as ungrammatical without additional effort. Accordingly, syntactically anomalous sentences may show increased patterns of activation reflecting post-linguistic repair processes, or rather, display minimal patterns of activation reflecting early rejection—at the verb-object—by the parser. Support for “early rejection” would be obtained if most sentence types, but particularly indeterminate and pragmatically anomalous sentences, engage pragmatic resources more so than syntactically anomalous sentences. Support for a “repair” hypothesis, thus, would pattern these sentences with pragmatically anomalous and indeterminate sentences.

#### Method

##### Participants

Eighteen Concordia University students (14 females) participated in this experiment. They ranged in age from 18 to 38 years (*M* = 24.2), were native speakers of English, right-handed, and had normal (20/20 Snellen) vision. Participants gave informed consent and reported having no history of cognitive impairment or brain injury. They were compensated $60 for the session, which lasted approximately 90 min.

##### Materials

We employed the 648 sentences developed in our norming study, with the same distribution of materials into six lists, and the same fillers and questions.

##### Measures and apparatus

A 3 Tesla Whole Body MR System (MAGNETOM Trio, Siemens Medical Systems, and Erlangen, Germany) was used for image acquisition. Before the fMRI run, 160 3D FLASH structural images were acquired in slices of 1.2 mm thickness in the sagittal plane (256 mm × 256 mm) yielding a spatial resolution of 1 mm × 1 mm × 1.2 mm for the anatomical volume. Time to repetition (TR) for the anatomical scan was 2300 ms and time to echo (TE) was 2.99 ms. The whole brain fMRI scan employed an echo-planar imaging (EPI) sequence measuring the blood oxygenation level dependent (BOLD) signal. A total of 31 functional slices per volume were acquired for each subject, in each run. These slices, which were acquired in the transversal plane, interleaved and, in ascending order, were 3 mm thick, at an inplane resolution of 3 mm × 3 mm (matrix size 64 × 64) and in a field of view (FOV) of 192 mm × 192 mm, with a 0.75 mm gap between them in order to avoid cross talking. The spatial resolution of functional images was 3 mm × 3 mm × 3.75 mm. A complete scan of the whole brain was acquired in 2000 ms (TR); the flip angle was 75°, TE = 30 ms, and a total number of 550 volumes were acquired during one functional run. Each subject participated in two functional runs, for which we used the online automatic motion correction sequence, implemented prospective and retrospective by the scanner (MOCO series).

##### Procedure

**Figure 1:**
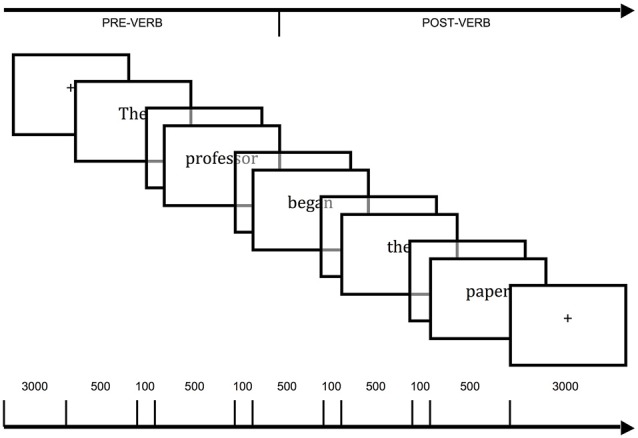
**Time course of stimuli presentation employed in the functional magnetic resonance imaging (fMRI) experiment.** Each word was presented for 500 ms with an interstimulus interval (ISI; blank screen) of 100 ms. The total intertrial interval (fixation point, “+”) was 6000 ms. The main data analyses took into account blood oxygenation level dependent (BOLD) signal change from “pre-verb” event (3000 ms of fixation point plus *The professor*) to “post-verb” event (which included the verb and complement, *began the paper*, plus 3000 ms of fixation point).

Sentences were presented visually on a projector placed at the head of the fMRI tunnel, which could be observed by the participants through a mirror placed on the head coil. A computer running E-prime (Schneider et al., 2002) was used for the presentation of stimuli and collection of Yes/No responses. Sentences were presented word-by-word in black text centered on a gray background. Each word was presented for 500 ms with an interstimulus interval (ISI) of 100 ms between words. Prior to each sentence, a fixation cross (+) was presented in the center of the screen for 6000 ms (see Figure 1). Responses to the filler questions were registered on a button box with buttons corresponding to the index finger and the middle finger for the Yes and No responses, respectively. Trials were divided into two randomized blocks. These blocks corresponded to two functional scans, each lasting approximately 18 min with a short break between them. This was followed by a 9-min structural scan. Prior to the scanning session, the participants were provided with practice trials on a laptop computer and were familiarized with fMRI scanning using a simulator.

##### Preprocessing of fMRI data

BrainVoyager QX (Brain Innovation B.V., Maastricht, Netherlands) software was used for fMRI data preprocessing and analysis. The functional bi-dimensional images of every subject were preprocessed to correct for the difference in time slice acquisition (slice scan time correction). In addition to linear detrending, a high-pass filter of three cycles per time course (frequency domain) was applied to the corrected 2D slices. The functional series was then preprocessed to correct for possible motion artifacts in any plane of the tridimensional space and to ensure that movements in any plane did not exceed 3 mm. The motion correction was performed in BrainVoyager by an algorithm which aligns the subsequent functional volumes to the first one. Then, the six movement parameters (three translations, three rotations) are displayed graphically, thus allowing us to verify the magnitude of the movement during the scanning session. In our case, no subject moved their head more than 3 mm or 3° along or around any of the three spatial axes. As for co-registration and spatial normalization, we employed the standard procedure in BrainVoyager. To this end, the functional volume was first spatially aligned with the raw anatomical volume in two steps: (1) an initial alignment based on the information stored in the header of the DICOM file about the position of the slices relative to the center of the scanner; and (2) a fine tuning or rigid body alignment which was done manually by controlling the translation and rotation of the functional volume relative to the anatomical one (see Goebel et al., 2006). Later, these functional images were used to reconstruct the 3D functional volume for every subject and every run. The 3D functional volume was aligned with the corresponding 3D anatomical volume, and both were normalized to standard Talairach space (Talairach and Tournoux, 1988) and to an isovoxel resolution of 1 mm × 1 mm × 1 mm. Spatial smoothing using a Gaussian kernel at 8 mm full width at half maximum (FWHM) was applied to the 3D functional data.

##### Statistical analyses of fMRI data

We used a rapid event-related block design to analyze our data. The time-course of each trial was separated in two periods, pre-verb and post-verb (Figure 1). The latter 3000 ms of the fixation presentation and the subject NP in the sentence comprised the pre-verb event (e.g., 3000 ms of “+” and “*The professor”*). The verb and its NP complement, as well as the first 3000 ms of the next fixation presentation comprised the post-verb event (e.g., “*began the paper*” and 3000 ms of “+”). These pre-verb and post-verb intervals were then convolved with the two Gamma HRF’s (onset of response = 5 s, undershoot = 16 s, dispersion = 1 s, response to undershot ratio = 6) as implemented by default in BrainVoyager. For each sentence type, a pre-verb and post-verb predictor was included as fixed-factors in a single subject General Linear Model (GLM). The parameters of this GLM model were subsequently entered into a second random-effect GLM model used for group analysis (Penny and Holmes, 2003). The rationale behind this contrast method was to isolate processes underlying the combination of a verb and its complement as well as post-linguistic processes that might be triggered by the sentence. In a first analysis, activation maps for the entire brain were computed voxelwise at the group level for the contrast measuring the difference in BOLD signal change from the pre-verb to the post-verb level of activation for each of the six sentence types. The criteria used to display the activation maps for the post-verb > pre-verb contrasts were: (a) a statistical threshold of at least *p* < 0.001 at the voxel level, which also corresponded to a false discovery rate correction of q(FDR) < 0.05; and (b) a cluster size of at least 108 adjacent significant voxels (108 mm^3^). In addition, for each sentence type we computed a laterality index reflecting the proportion of the difference between RH and LH voxels activated significantly above the threshold, relative to the sum of both RH and LH voxels using the formula ((#right − #left)/(#right + #left)). We predicted that a lateralization index for each of the sentence types would help us understand the resources engaged in processing the range of syntactic and semantic conditions we employed and thus contribute to a broader picture of the neuronal correlates of sentence processing.

In order to identify the neuronal substrates engaged specifically in processing indeterminate sentences in contrast with other sentence types, we employed a second analysis with the following procedure: (a) the areas identified in the first analysis for the indeterminate sentence type were defined as regions of interest (ROIs); and (b) in each of these ROIs we assessed the number of voxels for which the difference between predictors (post-verb > pre-verb) was significantly higher for the indeterminate than for other sentence types; for this analysis we employed the same statistical threshold (*p* < 0.001). This second analysis allowed us to obtain a map of neurological resources employed in the interpretation of indeterminate sentences throughout the brain, while simultaneously investigating the engagement of these areas in the interpretation of other sentence types with regards to the post-verb > pre-verb contrast.

#### Results and Discussion

The contrast of interest (post vs. pre-verb) indicates that each sentence type activates a large network of clusters in both hemispheres (for the full set of significantly activated regions per condition, see Table 4). In addition to the more traditional “language areas” (i.e., Broca’s and Wernicke’s), we observe that several regions emerge systematically across sentence types, such as the medial prefrontal cortex (e.g., ACC), and regions in the superior parietal lobule and temporal lobe, bilaterally.

**Table 4:** **Activated regions for all sentence conditions**.

Left hemisphere regions	Talairach coordinates			Volume (mm^3^)	Maximum *t*_(17)_	Right hemisphere regions	Talairach coordinates			Volume (mm^3^)	Maximum *t*_(17)_
**Preferred**						**Preferred**
Insula	−31	22	2	167	4.59	Fusiform gyrus (BA 37)	40	−51	−15	479	4.90
Postcentral gyrus (BA 40)	−29	−35	54	119	4.32	Lingual gyrus (BA 17)	20	−94	−10	213	4.62
Precentral gyrus (BA 6)	−44	−6	47	569	4.92	Medial frontal gyrus (BA 6)	1	0	50	2033	5.03
Sub-gyral (BA 20)	−38	−16	−18	162	4.83	Middle occipital gyrus (BA 19)	45	−75	−6	460	4.86
Thalamus (Medial	−7	−17	8	2071	5.43	Thalamus	13	−17	12	194	4.80
Dorsal Nucleus)
**Non-preferred**						**Non-preferred**					
Claustrum	−29	16	5	325	4.61	Insula (BA 13)	33	17	6	537	5.13
Inferior parietal lobule (BA 40)	−53	−43	24	1193	5.23	Middle occipital gyrus (BA 19)	46	−76	−6	1073	6.34
Medial frontal gyrus (BA 6)	−5	−4	54	179	4.50	Superior frontal gyrus (BA 46)	4	12	55	150	4.87
Middle frontal gyrus (BA 46)	−43	15	23	3060	5.59	Superior parietal lobule (BA 7)	30	−55	43	205	4.58
**Full-VP**						**Full-VP**					
Cingulate gyrus (BA 32)	−13	17	23	2311	7.31	Cingulate gyrus (BA 24)	11	12	31	2250	8.07
Inferior frontal gyrus (BA 45)	−39	21	14	1143	5.07	Cingulate gyrus (BA 24)	20	−12	42	231	5.36
Inferior parietal lobule (BA 40)	−38	−36	40	492	4.80	Claustrum	26	24	5	245	5.11
Middle temporal gyrus (BA 39)	−48	−57	10	265	4.78	Claustrum	31	13	0	253	4.90
Precentral gyrus (BA 4)	−18	−21	54	534	5.22	Lentiform nucleus	11	3	3	922	5.47
						(Lat. Globus Pallidus)
Precentral gyrus (BA 6)	−38	−13	61	128	4.53	Lingual gyrus (BA 17)	17	−89	−4	258	4.59
Superior frontal gyrus (BA 6)	−1	5	63	2707	6.34	Middle frontal gyrus (BA 6)	37	−5	50	142	4.57
Thalamus (Medial	−5	−12	10	1963	5.52	Superior temporal gyrus (BA 22)	54	−29	3	117	4.98
Dorsal Nucleus)						Supramarginal gyrus (BA 40)	34	−42	30	375	5.32
**Indeterminate**						**Indeterminate**					
Fusiform gyrus (BA 19)	−26	−77	−11	748	6.01	Fusiform gyrus (BA 37)	42	−50	−10	115	4.42
Inferior parietal lobule (BA 40)	−36	−36	38	171	4.57	Insula (BA 13)	35	14	7	1467	5.46
Insula (BA 13)	−34	14	8	845	4.90	Superior parietal lobule (BA 7)	31	−54	40	856	4.62
Middle temporal gyrus (BA 22)	−53	−36	7	5396	7.25	Superior temporal gyrus (BA 22)	48	−23	1	700	4.97
Middle temporal gyrus (BA 39)	−53	−71	11	150	5.03	Medial frontal gyrus (BA 6/BA 32)	0	12	42	1584	5.97
Posterior cingulate (BA 23)	−1	−31	23	170	4.97	Thalamus (Medial Dorsal Nucleus)	9	−19	9	897	5.44
Precentral gyrus (BA 44)	−49	5	9	431	5.04						
Precentral gyrus (BA 6)	−50	−2	30	120	4.75						
Superior frontal gyrus (BA 10)	−31	57	24	564	5.14						
Superior parietal lobule (BA 7)	−23	−67	41	367	4.74						
Superior temporal gyrus (BA 38)	−54	7	−7	512	6.14						
Thalamus	−9	−21	2	4288	7.08						
**Syntactically anomalous**						**Syntactically anomalous**					
Insula (BA 13)	−35	16	12	3214	6.61	Fusiform gyrus (BA 37)	42	−52	−16	739	7.63
Middle occipital gyrus (BA 18)	−28	−79	−10	126	4.33	Inferior occipital gyrus (BA 19)	45	−73	−5	568	5.51
Superior temporal gyrus (BA 21)	−52	−17	−3	235	4.97	Insula (BA 13)	31	18	11	592	5.16
Thalamus (Medial Dorsal Nucleus)	−8	−14	8	143	4.56	Precuneus (BA 7)	22	−56	34	1088	5.88
						Thalamus (Ventral Lateral Nucleus)	10	−9	8	886	5.33
**Pragmatically anomalous**						**Pragmatically anomalous**					
Insula (BA 13)	−35	14	12	381	4.77	Angular gyrus (BA 39)	32	−61	33	159	4.82
Superior frontal gyrus (BA 6)	−1	21	61	403	5.52	Insula (BA 13)	34	16	11	1208	6.09
Thalamus	−7	−30	−2	142	4.29	Medial frontal gyrus (BA 32)	3	7	43	4818	6.99
						Middle occipital gyrus (BA 19)	49	−75	−8	168	4.54

**Figure 2:**
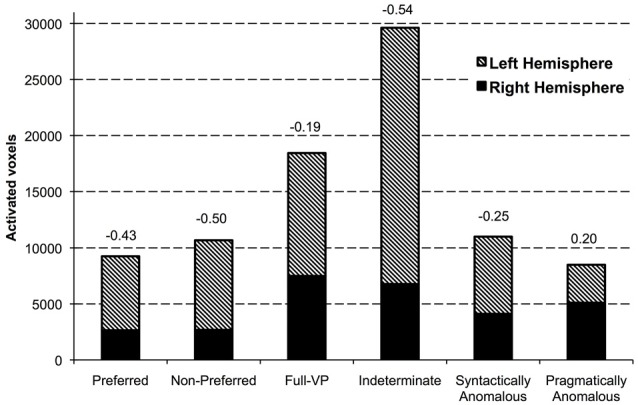
**Volume of activation (1 voxel = 1 mm^3^) surpassing the statistical (*t*_(17)_ > 3.97, *p* < 0.001) and spatial threshold (minimum 108 contiguous voxels in a cluster) for the contrast postverb > preverb for each sentence type in the fMRI experiment.** The laterality index, expressed as the ratio of the difference between the number of voxels activated on the right vs. on the left relative to the total number of voxels, is displayed above each bar. Negative values indicate propotionately greater voxels on the left, whereas positive indicates proportionately greater voxels activated on the right hemisphere (RH).

In Figure 2, we present the volume of activation for left and right hemispheres surpassing the statistical (*p* < 0.001) and spatial (108 mm^3^ contiguous voxels) thresholds, as well as the values of the laterality index. This index reveals that, with the exception of the pragmatically anomalous sentence type, which had a positive value indicating more RH than LH activation, all others had negative values, showing a predominance of LH over RH activation. In addition, indeterminate sentences showed a higher volume of activation in the RH compared to all other conditions with the exception of the full-VP sentences. Indeterminate sentences also showed a greater spread of activation in the LH—and in fact across the whole brain—relative to all other sentence types.

The general picture that emerges from this descriptive analysis is consistent with previous findings vis-à-vis the lateralization of linguistic and non-linguistic resources involved in sentence interpretation. In addition, these findings suggest that indeterminate sentences recruit computational resources that surpass those involved in the interpretation of both canonical and anomalous constructions, with both linguistic and non-linguistic processes contributing to interpretation. The novelty of our (post-verb > pre-verb) approach is that it isolates verb-object noun composition as the source of neuronal activity for each sentence type. This method specifically addresses how the different verb-noun combinations affect interpretive processes, permitting us to isolate neurological structures associated uniquely with indeterminacy.

**Figure 3:**
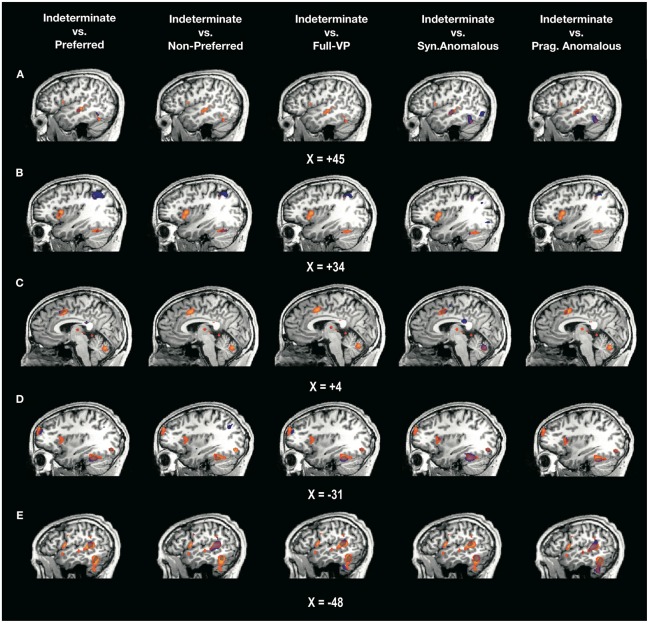
**Overlap between two statistical maps.** Depicted in orange are the regions significantly activated by the contrast postverb > preverb for the indeterminate sentences (minimum *t*_(17)_ = 3.97, *p* < 0.001, *FDR* < 0.05). Depicted in blue are the voxels for which the difference in activation postverb > preverb is significantly greater in indeterminate compared to each of the other sentence types. The overlap between the two statistical maps (in purple) corresponds to the voxels that are specific to the processing of the composition between verb and complement in the indeterminate vs. each of the other sentence types. *X* values indicate coordinates in Talairach space: **(A)** right temporal lobe; **(B)** right lateral prefrontal cortex; **(C)** medial prefrontal lobe; **(D)** left lateral prefrontal cortex; **(E)** left temporal lobe. For a high-resolution version of this figure, see Supplementary Material.

Figure 3 displays two overlapping activation maps, one showing the significant regions of activation for indeterminate sentences (all *t*’s > 3.97, all *p*’s < 0.001) and the other showing the specific voxels for which indeterminate sentences elicited significantly greater activation than the contrasting sentence types (all *t*’s > 3.97, all *p*’s < 0.001). Based on the network activated by the contrast post-verb > pre-verb for indeterminate sentences in the whole brain, we selected five ROIs as being particularly informative for demarcating the resources recruited specifically for indeterminate sentence comprehension relative to the other sentence types. These regions were the STG and the IFG bilaterally, as well as the ACC (Table S1 in Supplementary Material shows all contrasts).

In the R-STG, indeterminate sentences yielded statistically higher activation than preferred, syntactically anomalous and pragmatically anomalous sentence types. However, indeterminate sentences did not yield higher activation relative to the non-preferred and full-VP sentences. More specifically, from a total volume of 700 voxels that surpassed the statistical threshold in the R-STG, there were 74 voxels that were significantly more activated for indeterminate sentences compared to preferred sentences (average *t*-value for this difference: *t*_(17)_ = 4.13, *p* < 0.001) and 97 voxels that were significantly more activated for indeterminate sentences compared to pragmatically anomalous sentences (average *t*-value for this difference: *t*_(17)_ = 4.13, *p* < 0.001).

In the R-IFG, indeterminate sentences also yielded statistically higher activation than preferred in 128 voxels (average *t*-value: *t*_(17)_ = 3.99, *p* < 0.001). No other comparisons at this ROI were significant. These results are consistent with the hypothesis that indeterminate sentences might engage greater RH resources than preferred sentences, and also consistent with studies that show R-IFG activation for metonymic sentences (e.g., *Africa is hungry*, Rapp et al., 2011), which arguably require pragmatic processes for recovering the proper interpretation of the subject noun. However, indeterminate sentences did not differ from non-preferred and full-VP sentences, suggesting that as constructions deviate from the canonical form, they show attenuated margins of differentiation with the indeterminate condition. In the ACC, indeterminate sentences elicited greater activity than preferred (584 voxels, average *t*-value: *t*_(17)_ = 4.39, *p* < 0.001), syntactically anomalous, and pragmatically anomalous (946 voxels, average *t*-values: *t*_(17)_ = 4.30, *p* < 0.001) constructions.

Indeterminate sentences yielded greater activation in L-IFG (Broca’s area) than syntactically and pragmatically anomalous conditions, but no differences were found at this ROI between indeterminate and the three other sentence types—preferred, non-preferred and full-VP. One possible interpretation of these results is that the two anomalous conditions engage less so the L-IFG because they require different forms of repair. In the case of the syntactically anomalous condition, it is possible that an early rejection of its ungrammatical verb-complement combination requires little effort from the parser. As for the pragmatically anomalous condition, the greater involvement of RH regions, as shown by our laterality indices, might suggest that repair of these sentences lies beyond Broca’s area.

A total of 169 voxels in the L-IFG showed more activation for the contrast post-verb > pre-verb in indeterminate relative to pragmatically anomalous sentences (average *t*-values: *t*_(17)_ = 4.18, *p* < 0*.001*). In the left L-STG (Wernicke’s area), indeterminate sentences showed significant differences relative to all sentence types (all *p*’s < 0.001). These results complement the findings from the volumetric and laterality descriptive analyses by showing that processing of indeterminate sentences relies on both a greater volume and a higher level of activity in both LH and RH compared to all other sentence types, including those that are syntactically and pragmatically anomalous.

**Figure 4:**
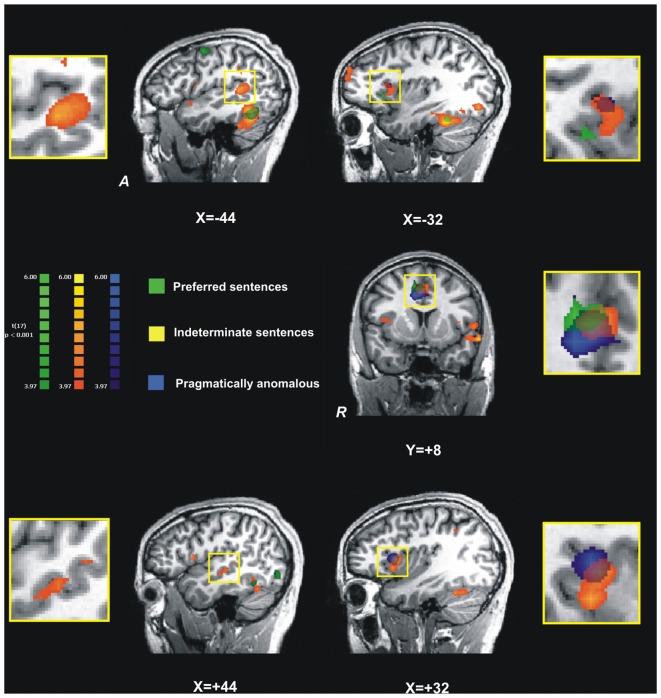
**The overlap and spatial differences between activation maps (changes in BOLD signal) for the three main sentence conditions, preferred (green clusters), indeterminate (orange clusters), and pragmatically anomalous (blue clusters) in five regions-of-interest.** Left STG and IFG are shown in top row; the anterior cingulate cortex (ACC), in the middle; and right STG and IFG in the bottom row. In the left and right STG we observe a greater activation for indeterminate sentences, with no overlap with the other sentence types; however, in the IFG, both on the right and left there is overlapping with preferred sentences. In the left IFG (top right) there is greater activation for indeterminate (BA 44) but overlapping with pragmatically anomalous (see insert); a ventral region of BA 44 shows greater sensitivity for preferred sentences. In ACC we observe overlapping activations, with indeterminate sentences activating a more anterior area, while preferred and pragmatically anomalous sentences activate more the posterior parts of ACC; all activated regions in the ACC are more right-lateralized. In the right STG we observe significant activation for indeterminate sentences only, and in the right IFG we note common and distinct activations for both pragmatically anomalous and indeterminate sentences but not preferred. *X* and *Y* values indicate coordinates in Talairach space. ACC, anterior cingulate cortex; IFG, inferior frontal gyrus; STG, superior temporal gyrus.

Our final analysis focused on the spatial overlap of the activation maps for indeterminate, preferred and pragmatically anomalous sentences in the five prefrontal and temporal ROI (IFG and STG bilaterally and ACC; see Figure 4). For this analysis we focused on these three sentence types with different but complementary motivations. First, the preferred sentence is the most common type of control sentence employed in all experimental studies on indeterminacy, including the two neuroimaging studies we reviewed. Second, the pragmatically anomalous condition would allow us to see how it patterns with the indeterminate condition, thus giving us a better understanding of the difference between pragmatic anomaly and indeterminacy, in the ROI. A spatial intersection of the three sentence types was observed in the ACC. However, in the L-IFG, the statistical maps of indeterminate and pragmatically anomalous sentences were spatially distinct from that of preferred sentences although they overlapped with each other. Moreover, in the R-IFG the activation map for preferred sentences did not surpass the statistical threshold, whereas there was an overlap between pragmatically anomalous and indeterminate sentence types, which surpassed the threshold. Finally, in the temporal lobes, we observed an overlap of indeterminate and preferred statistical maps in the STG but, surprisingly, no significant activation for pragmatically anomalous sentences.

This pattern of activation suggests that indeterminate sentences share properties of both canonical and pragmatically anomalous sentences. On the one hand, indeterminate and preferred constructions mutually activate the STG bilaterally, suggesting that the two sentence types may undergo a qualitatively similar semantic analysis, consistent with studies showing bilateral STG activation for canonical sentences (e.g., Friederici et al., 2003). On the other hand, indeterminate sentence activation diverged from that of preferred sentences and instead patterned with pragmatically anomalous sentences in the R-IFG, suggesting that beyond semantic analysis, indeterminate sentences may be pragmatically distinct from canonical sentence types.

## General Discussion

The present study provides new evidence for the neurological underpinnings of the process of indeterminate sentence interpretation. We found that indeterminate sentences engage a wide network involving left, right, and medial-frontal regions of the brain. More specifically, compared to preferred sentences, indeterminate sentences required greater involvement of R-IFG (BAs 44, 45), bilaterally the STG (primarily BA 22) as well as the ACC (BAs 24, 32). In addition, our results also show that indeterminate sentences patterned with our pragmatically anomalous sentences (*The author drank the book*), but elicited greater hemodynamic changes in all ROI. Finally, our laterality indices show greater involvement of LH regions for all sentence types, except for the pragmatically anomalous condition. However, indeterminate sentences elicited greater activation in RH regions relative to all control sentences, except the full-VP condition.

Overall, what emerges from our study is that indeterminate sentences engage more areas than control sentences (see Table 4) and do so with greater intensity at all ROIs (Figure 4). More importantly, we show that indeterminate sentences engage areas consistent with a pragmatic view of indeterminate sentence resolution. In the following section, we discuss our fMRI findings in relation to several of our hypotheses and contrast them with the cognitive neuroscience literature on coercion effects.

### Coercion or Pragmatic Processes?

The primary goal of our study was to obtain a general activation map for indeterminate sentences. Given the inconsistent results found in other studies (Pylkkänen and McElree, 2007; Husband et al., 2011), we wanted to establish which neuroanatomical resources are deployed in the resolution of indeterminacy. But in contrast with these other studies, which were devised to find the neuroanatomical source of *coercion*, we were also interested in contrasting two hypotheses on how indeterminacy might be resolved (or attempted to be resolved)—whether by semantic processes of coercion said to be localized primarily at the vmPFC or at the L-IFG, or whether by a wider network thus characterizing pragmatic processes.

Overall, our results show that indeterminate sentences recruit a vast network involving both temporal and frontal regions bilaterally. Although indeterminate sentences—consistent with linguistic processes—are clearly left-lateralized, the alleged mismatch between verb and NP complement in cases such as *The author started the book* recruits more RH resources than do preferred sentences (…*wrote the book*). In addition, our results also show that the activation maxima for indeterminate sentences occur in a more dorsal area of the L-IFG compared to preferred sentences, suggesting potential differences in the syntactic properties of these sentences (see, e.g., de Almeida and Dwivedi, 2008). Interestingly, Husband et al. (2011) also found significant involvement of the L-IFG for indeterminate sentences, thus, indeed this region appears to play a critical role in indeterminate sentence processing. In contrast with our position, however, Husband et al. (2011) proposed that the difference between indeterminate and preferred sentences in the L-IFG reflects the extra compositional mechanisms required by coercion. They suggest that the L-IFG works in the detection of the mismatch between verb and noun complement and also that it “may function to select and retrieve the noun’s event-related meaning” (p. 3262). Although we cannot rule out this interpretation, it is also possible that this difference instead reflects the syntactic complexity of indeterminate sentences. As we have proposed elsewhere (see de Almeida and Dwivedi, 2008; de Almeida and Riven, 2012), indeterminate VPs may include a structural gap which triggers the enrichment process and may, therefore, elicit unique activations in the L-IFG. It is also our suggestion that this gap might be the source of “coercion effects”, namely the RT differences between indeterminate and control sentences found in some behavioral studies (de Almeida and Dwivedi, 2008). Whether or not the L-IFG activation reflects coercion or syntactic complexity will ultimately be settled by neuroimaging experiments designed specifically to contrast these positions.

While Husband et al.’s (2011) fMRI results—which focused on activation in the L-IFG—challenged the MEG localization data (Pylkkänen and McElree, 2007), our results, obtained on the basis of an initial whole brain analysis, have corroborated in part those MEG findings. In fact, we propose that our spatial data complement those temporally sensitive MEG findings and converge with regards to the nature of indeterminate sentence processing. In particular, the MEG study showed that indeterminate sentences produced a bilateral temporal activation, followed by activation that was source-localized at ventromedial frontal areas (Pylkkänen and McElree, 2007). Similarly, we found bilateral STG activation with corresponding bilateral activation in the IFG and medial activation in the ACC. Thus, collectively these findings suggest that indeed indeterminate sentences engage a neural network of left, right, and medial regions, with activation unfolding first in the LH, then the RH, and finally in medial regions of the network.

If indeed each of these regions participates in the resolution process, this challenges a view of indeterminacy that links the resolution uniquely to the L-IFG as Husband et al. (2011) proposed. Consequently, this casts doubt on a version of coercion theory which attributes the resolution of these sentences strictly to a semantic coercion operation. Instead, these empirical observations corroborate a pragmatic theory of indeterminacy resolution. Consistent with data from other studies involving canonical as well as anomalous constructions (for reviews see Lau et al., 2008; Friederici, 2011), we propose that indeterminate sentence processing appears to initially undergo a linguistic, denotational analysis, which then triggers pragmatic resolution processes (a search for an intended message). Interestingly, it appears that other forms of coercion also undergo a pragmatic resolution after an initial literal interpretation. In a MEG study investigating aspectual coercion (*Throughout the day the student sneezed…*). Brennan and Pylkkänen (2008) found that the mismatch between a punctual event (*sneeze*) and a durative context (*throughout the day*), elicits a RH activation about 340 ms after the verb. This RH activation is followed by an AMF at around 450 ms. This two-stage model of the aspectual mismatch resolution is preceded by an initial semantic composition that is only later deemed anomalous, thus requiring pragmatic repair—so that *sneeze* can be interpreted iteratively.

Although our study was not designed specifically to replicate Pylkkänen and McElree’s (2007) MEG findings—and in fact we adopt a different theoretical perspective—both their MEG and the present fMRI results point in a similar direction with regards to the neuroanatomic signature of indeterminate sentence interpretation: activation spreads from left to right to medial regions of the brain. The only exception to the convergence between these two studies is that we found medial activation in the ACC as opposed to the vmPFC. A possible explanation for this disparity may be that the ACC is the anatomical source of the AMF effect reported by Pylkkänen and McElree (2007). Because the ACC is anatomically (and, by hypothesis, functionally) connected to the vmPFC (Margulies et al., 2007) it is possible that the MEG effects reflect the spread of activation from ACC into pre-frontal areas. As has been shown in several studies, the cingulate cortex is involved not only in managing conflict resolution, but also in language comprehension tasks such as judgment of discourse coherence (Virtue et al., 2006), detection of semantic/pragmatic anomaly (e.g., Ni et al., 2000), drawing inferences from text (Ferstl et al., 2008) and the interpretation of non-literal expressions such as metaphors (e.g., Bottini et al., 1994; Bambini et al., 2011). Thus, the ACC appears to be involved in diverse types of pragmatic-level computations, above and beyond the processing of determinate sentences. Given the comparably better spatial resolution of fMRI over MEG, and the fact that vmPFC activation was not found in ours or Husband et al.’s (2011) fMRI studies, we suggest that Pylkkänen and McElree’s (2007) medial activation likely reflects ACC processes.

In summary, the present fMRI results point to a neuroanatomical source of coercion effects compatible with a pragmatic view of indeterminate sentence interpretation (e.g., de Almeida, 2004; de Almeida and Dwivedi, 2008). The widespread activations found for indeterminate sentences suggest that many sources of information might be required to attempt to resolve indeterminacy arising from potential verb-complement mismatches. Our data are in conflict with Husband et al. (2011) for whom “the mismatch and its repair only affect semantic composition and do not recruit other processes for repair or rejection” (p. 3262). In fact, we show that many other resources are recruited to resolve sentence indeterminacy. More in line with our view are results from two ERP studies which, nonetheless, take a different theoretical approach. Kuperberg et al. (2010) propose that coercion effects might be due to “implicit attempts to retrieve relevant information from semantic memory to ‘fill-in’ such mismatches” (p. 2698). Further, Baggio et al. (2010) propose a “unification” approach according to which a verb’s semantic-structural representation calls for an event complement while discourse provides a filler event. These ideas are in fact compatible with the pragmatic model we support—including the suggestion by de Almeida and Dwivedi (2008) that if there is a processing difference between indeterminate and control sentences it might be due to inferential processes called for by a structural VP gap inherent in indeterminate constructions. Although the present study did not aim to investigate specifically the VP gap in indeterminate sentences, their greater—but spatially distinct—activation of L-IFG compared to control sentences suggests the possibility that if there is a gap it might be responsible for triggering pragmatic processes that enrich the initial representation of the sentence.

It is important to note that pragmatic enrichment triggered by a syntactic gap (*…begin* [*vp-gap*] *the book*) differs from semantic interpolation (…*begin [to write] the book*). The latter assumes that the *enriched* semantic composition is parasitic on a suitable *event* to resolve the hypothetical verb-object mismatch of indeterminate sentences. In contrast, pragmatic enrichment triggered by a VP gap occurs beyond semantic composition and is characterized by searching—possibly abductively—for the intended meaning of an indeterminate expression. Moreover, a gap thus conceived is not a “silent verb” (Pylkkänen, 2008) but simply a syntactically determined position; as such, it works as a trigger for *inferences*, not for actual verb fillers.

### The Neurological Underpinnings of Semantic and Pragmatic Processes

If coercion effects are indeed *pragmatic* effects reflecting attempts to resolve indeterminacy, as we suggest, we are left with a view of semantic composition that contrasts with the enriched form of compositionality as proposed by Pustejovsky (1995) and others (see also Jackendoff, 1997; Traxler et al., 2005). Semantic composition might be a process that relies primarily on lexical denotations and syntactic computations—possibly served by left temporal and frontal structures, as suggested by neuroimaging and brain recording studies (e.g., Vandenberghe et al., 2002; Lau et al., 2008; Makuuchi et al., 2009; Friederici, 2011), with extra computational pragmatic resources being tapped depending on the degree of specificity of the resulting verb-internal composition. Thus conceived pragmatic computations are not constitutive of sentence meaning but a consequence of sentence indeterminacy. According to this perspective, indeterminate sentences are *fully compositional*: a sentence such as *The author started the book* is true or false if the author started doing something, regardless of what the author started doing.

Thus far, no studies have provided conclusive evidence for coercion *per se*, but have rather shown that indeterminate sentences yield a processing cost. Moreover, there is no evidence that processing costs reflect *linguistic* analyses culminating in an enriched form of semantic composition. While processing costs can be accounted for by diverse theoretical approaches (see de Almeida and Dwivedi, 2008; Baggio et al., 2010; de Swart, 2011; Dölling, 2014), we have rather suggested that they might be a manifestation of widespread activations compatible with a pragmatic account of indeterminacy resolution. The volume of activation surpassing our statistical and spatial thresholds in both hemispheres obtained for indeterminate sentences may indicate that indeterminacy resolution is not bound by *linguistic* computations. Rather, it may be that the involvement of left, right and medial cortical structures reflect a conflict between diverse sources of information—occurring beyond linguistic composition—as to what type of event *The author started the book* refers to.

A standing issue with regards to the semantics-pragmatics interface is the division of labor between computations that are linguistic, mandatory and those that are subject to extra-linguistic contextual factors. Coercion theory assumes that an alleged verb-noun mismatch requires extra semantic computations. The view we articulate—which is in fact compatible with that of others (e.g., Egg, 2005; Dölling, 2014)—is that the resolution of indeterminacy is a pragmatic, extra-linguistic process. Akin to the process of interpreting a metaphor, an indeterminate sentence might trigger pragmatic computations as an attempt to calculate what is intended by the speaker (Grice, 1989). Our indeterminate sentences patterned with pragmatic-violated sentences—engaging left, right, and medial regions—showing that indeed their interpretative processes overlap substantively with sentences that demand a pragmatic resolution.

However, despite their analogs patterning overall, critical differences emerged in the activation of these two sentence types, with indeterminate sentences activating a much broader cortical network. This greater activation suggests that neurological resources involved in sentence interpretation are sensitive to differences between these sentence types. A sentence such as *The author drank the book*, violates our common understanding of what the verb *to drink* means and the type of complement it may take (selectional restrictions), but it carries no true indeterminacy. In principle, we know what the subject (*The author*) did with the object (*the book*)—namely, drank it. A plausible way of interpreting this sentence would be by rejecting a literal, fully compositional meaning and understanding it as a metaphor (consider, for instance, an author seeking to quench her thirst for knowledge). An indeterminate sentence, however, does not call for the rejection of a literal interpretation but rather builds upon it. Although also fully compositional, determining what *The author* actually started doing with *the book* might call for an event that is obtained only by appealing to abduction, thus not by mandatory semantic processes. The greater whole-brain activation found for indeterminate sentences might reflect the greater state of uncertainty as to what these sentences convey.

Finally, two other contrasts are informative with regards to sentence processing mechanisms more generally. First, our syntactically and pragmatically anomalous sentences showed dissociative patterns of activation as indicated by the laterality index. Whereas pragmatically anomalous sentences showed greater RH than LH activation, syntactically anomalous sentences showed the reverse pattern. In addition, when compared to one another, the absolute volume of activation was greater in the LH for syntactically anomalous sentences and greater in the RH for pragmatically anomalous sentences. These differences in activation might reflect possibly different interpretation strategies that are triggered when the parser encounters syntactic vs. pragmatic violations. Specifically, the diminished activation in the RH for syntactically anomalous relative to pragmatically anomalous as well as indeterminate sentences, suggests the possibility that syntactic violations are rejected by the syntactic parser, whereas the other sentence types trigger more effortful repair processes. Second, perhaps the most unexpected result of our experiment was the spread of activation for full-VP sentences in the RH. In principle, full-VP sentences leave little open for further pragmatic interpretation, and accordingly we predicted that they would pattern with preferred sentences rather than indeterminate sentences in the RH. However, the opposite trend was observed. Notably, the post-verb > pre-verb contrast method we employed sets full-VP sentences apart from the other conditions specifically in terms of their semantic-compositional complexity. The combination between aspectual and event verbs in full-VP sentences may trigger other computations at the linguistic-cognitive interface. For instance, *evidentiality* (the information on the evidence for the proposition expressed), *aspect* (whether an event has marked point in time) and *tense*, interact in ways that are yet to be determined. Full-VP sentences together with indeterminate sentences, involve the imperfective aspect—i.e, they express an event that has no specific end point (*started*, *started writing*)—in contrast with our other, perfective sentence types (*wrote*, *read*, *yawned*, *drank*), which may call for different compositional and interpretive processes. There is also the possibility that full-VP sentences—which, as we mentioned, have greater *surface* complexity—can be considered somewhat convoluted, making a point that can be usually made with indeterminate sentences, within supporting discourse contexts. In this regard, there is an account of full-VP sentences that may be compatible with Grice’s theory: while it is clear that indeterminate sentences might call for enrichment, which we deem to be inferential, full-VP sentences may violate a Gricean maxim, “be perspicuous”. By failing to be succinct, these sentences say more than what is usually necessary to make a point. In other words, it may be the case that an indeterminate sentence such as *The author started the book* suffices to generate in the hearer the right interpretive inferences—ones that point to what is most commonly done with books by authors. Conversely, saying *The author started writing the book* may go beyond what is necessary to make the point, given the expectation that what authors usually do when they start books is write.

Yet another alternative is that full-VP sentences put the focus on the main event verb—*writing*—which may call for a presupposition: that the author had not started the book before. This may require conceptualizing a background upon which the presupposition can be anchored. Certainly, our study cannot dissociate between these alternatives, nor was it designed with that in mind. But it is clear from our results that full-VP sentences present linguistic and cognitive challenges that differentiate them from our other fully determined conditions. It is worth noting that the largest cluster of voxels activated for this condition was at the ACC (BA 24), overlapping with a large cluster activated for indeterminate sentences (BA 6/32; see Table 4), suggesting that a common challenge may underlie the interpretation of these sentences. One possibility is that activations at ACC reflect attempts at pragmatic resolution, which would be compatible with several alternative accounts of the full-VP effects laid out above.

On a final note, a major issue arising from the results of this study is how to account for the large clusters of LH activation associated with indeterminate sentences. We have assumed that the process of indeterminate sentence interpretation is obtained by pragmatic inferences, and in fact our prediction that RH and ACC structures would be more engaged in indeterminacy resolution was largely supported. We assume that the inferences that are triggered to resolve (or attempt to resolve) indeterminacy are computations over semantic/conceptual representations. They do not involve lexical items but their denotations, the concepts bearing on sentence meaning and beyond. It has been shown that the left temporal lobe is part of a “semantic network” which involves possibly categorically organized concepts in the temporal pole and extends posteriorly up to the supramarginal gyrus. As we discussed above, there is ample evidence for the role of the temporal poles in semantic processes (e.g., Damasio et al., 2004) and also evidence that the supramarginal gyrus and adjacent areas are involved in argument- and thematic-structure building (roughly, the assignment of roles to arguments of verbs to indicate who did what to whom; for review see de Almeida and Manouilidou, 2015). Our largest LH activation cluster was at the MTG, which is part of this network. This is compatible with results obtained by Husband et al. (2011). Given the posterior location of our cluster (BA 22/39) we postulate that its activation involves the building of the unusual argument/thematic structure that aspectual verbs such as *begin* engender.

Our second largest LH cluster for indeterminate sentences was at the thalamus. We are only beginning to understand the role that the thalamus plays in language comprehension and semantic processing, but there are indications that the thalamus is engaged in the detection of syntactic and semantic/pragmatic anomalies (Wahl et al., 2008), in lexical-semantic tasks, and in working memory supporting language comprehension (Crosson, 1999). The cluster of activation in the thalamus during indeterminate sentence processing was, in fact, the second largest of all clusters in the present study. Although this structure was activated to a lesser extent for other conditions, it seems to play a significant role in relaying different aspects of interpretation not only to the “semantic network” of the LH but to other regions, including the RH. Although *a priori* we did not expected thalamic effects to play a key role in the neuronal correlates we were investigating, one possibility presented by our data is that the thalamus becomes increasingly engaged in sentence processing when interpretation requires widespread coordination between diverse levels of linguistic and nonlinguistic computations.

### Conclusions

Overall, our results show that indeterminate sentences engage neurological substrates that go beyond those required to interpret determinate sentences—producing a hemodynamic response that is more compatible with a view that takes pragmatic inferences to be triggered beyond classical semantic composition. While nobody denies that inferential-pragmatic processes might be a consequence of earlier syntactic and semantic computations, the crux of the matter is what these syntactic and semantic computations yield. The coercion theory assumes that much of the process of indeterminate sentence interpretation should be resolved by earlier, mostly linguistic processes of type-shifting and semantic interpolation—with reduced pragmatic activity compared to determinate sentences. Our view, in contrast, is that the earlier linguistic computations leave indeterminate sentences unresolved, with a greater role played by pragmatic computations in search of what is intended.

Clearly, our data—as those of other neuroimating studies—cannot distinguish between hypotheses concerning the nature of indeterminate resolution without an understanding of the actual computations performed by activated neuronal tissue. It has proven difficult to reconcile neuroimaging and psycholinguistic data with linguistic-theoretical constructs aiming to understand how language representations and processes are implemented in the brain. In the case of neuroimaging studies, in particular, it has often been the case that neuroanatomical correlates of language processing have been hard to pinpoint due to numerous methodological variables (see, e.g., Indefrey and Cutler, 2004; Binder et al., 2009; Fedorenko and Kanwisher, 2009). Knowledge advancement in this domain naturally requires converging evidence, obtained through the application of diverse theoretical perspectives and methodological approaches. To date, however, only two studies have investigated how the interpretation of sentences such as *The author started the book* might be implemented in the brain, and both have been guided by the same theoretical position—that indeterminate sentences are resolved by semantic coercion. The present study provides new data on this phenomenon showing different neurological correlates, and suggests that, rather than semantic coercion, pragmatic computations play a dominant role in the interpretation of indeterminate sentences.

## Ethics Statement

Concordia University Human Research Ethics Committee (UHREC), Comite d’éthique de la Recherche (CER-IUGM). All participants gave informed consent, both for the behavioral normative task and for participation in the fMRI experiment.

## Author Contributions

RGA is the main author of the manuscript and the principal responsible for the conception and design of the study, theoretical background and general discussion; LR led the acquisition, analysis, and writing of the norming study, and contributed to fMRI data acquisition and analysis, and to manuscript editing; CM contributed to the design of the study, the preparation of materials, data acquisition and manuscript editing; OL led the analysis and reporting of the fMRI data, and contributed to manuscript editing; VDD contributed to the conception of the work, design and materials; GJ contributed to the design of materials and manuscript editing; BG contributed to the theoretical background and manuscript editing.

## Funding

This study was supported by grants from the Social Sciences and Humanities Research Council of Canada (SSHRC) and the Natural Sciences and Engineering Research Council of Canada (NSERC) to RGA and by a Major Collaborative Research Initiative grant from SSHRC to Gary Libben (director), Gonia Jarema, Eva Kehayia, Bruce Derwing, Lori Buchanan and RGA.

## Conflict of Interest Statement

The authors declare that the research was conducted in the absence of any commercial or financial relationships that could be construed as a potential conflict of interest.

## Footnotes

^1^A thorough review of the numerous theoretical positions on how semantic coercion, type coercion, or type shifting work is beyond the scope of the present article (see e.g., Partee, 1986; Briscoe et al., 1990; Goddard and Jayez, 1993; Pustejovsky, 1995; Jackendoff, 1997; for a review see also de Swart, 2011). Also, rather than following common practice and calling sentences such as those in (1) “coerced”—which is linked to one hypothesis about how they are semantically represented—we refer to them as *indeterminate* because there is uncertainty with regards to the state of affairs that the sentences denote, although truth-value judgments can be made (Gillon, 2004).

^2^We restrict our review to studies that have employed the same kinds of sentences as ours. Although other cognitive neuroscience studies were also concerned with the general phenomenon of coercion, they either employed other sentence types—which might involve different linguistic processes (e.g., Pylkkänen et al., 2009)—or employed techniques such as ERP (Baggio et al., 2010; Kuperberg et al., 2010) which does not allow for similar neuroanatomical predictions as ours (for a study with aphasics, see also Piñango and Zurif, 2001). ERP studies, in particular, have obtained similar results as the behavioral studies they followed, with different wave patterns for indeterminate sentences compared to control sentences. We address ERP effects in the “General Discussion” Section, below.

^3^It should be noted that some studies have shown equal involvement of both hemispheres in metaphor comprehension (Rapp et al., 2007; Stringaris et al., 2007), perhaps reflecting parallel or competing literal and metaphorical interpretations.

^4^We refer to these sentences as “pragmatically anomalous” because, although they contain what are regarded as *semantic* (e.g., selectional restrictions) violations, they might be interpretable by appealing to pragmatics. As Grice (1989) puts it, a speaker can blatantly violate a conversational maxim—such as to express an obvious falsity—in order to generate implicatures in the mind of the hearer. Akin to common metaphors (e.g., *My lawyer is a shark*), semantic ill-formedness might be used to trigger abductive processes of interpretation.

^5^This alpha level represents the adjusted threshold of statistical significance for pairwise contrasts that yield a 0.05 family-wise type I error rate, following the guidelines outlined in Kline (2004).
