# Supplementary figures and images for: The Neuronal Correlates of Indeterminate Sentence Comprehension: An fMRI Study
Source: Front Hum Neurosci. 2016 Dec 20;10:614. doi: 10.3389/fnhum.2016.00614 (PMC5168646; doi:10.3389/fnhum.2016.00614)

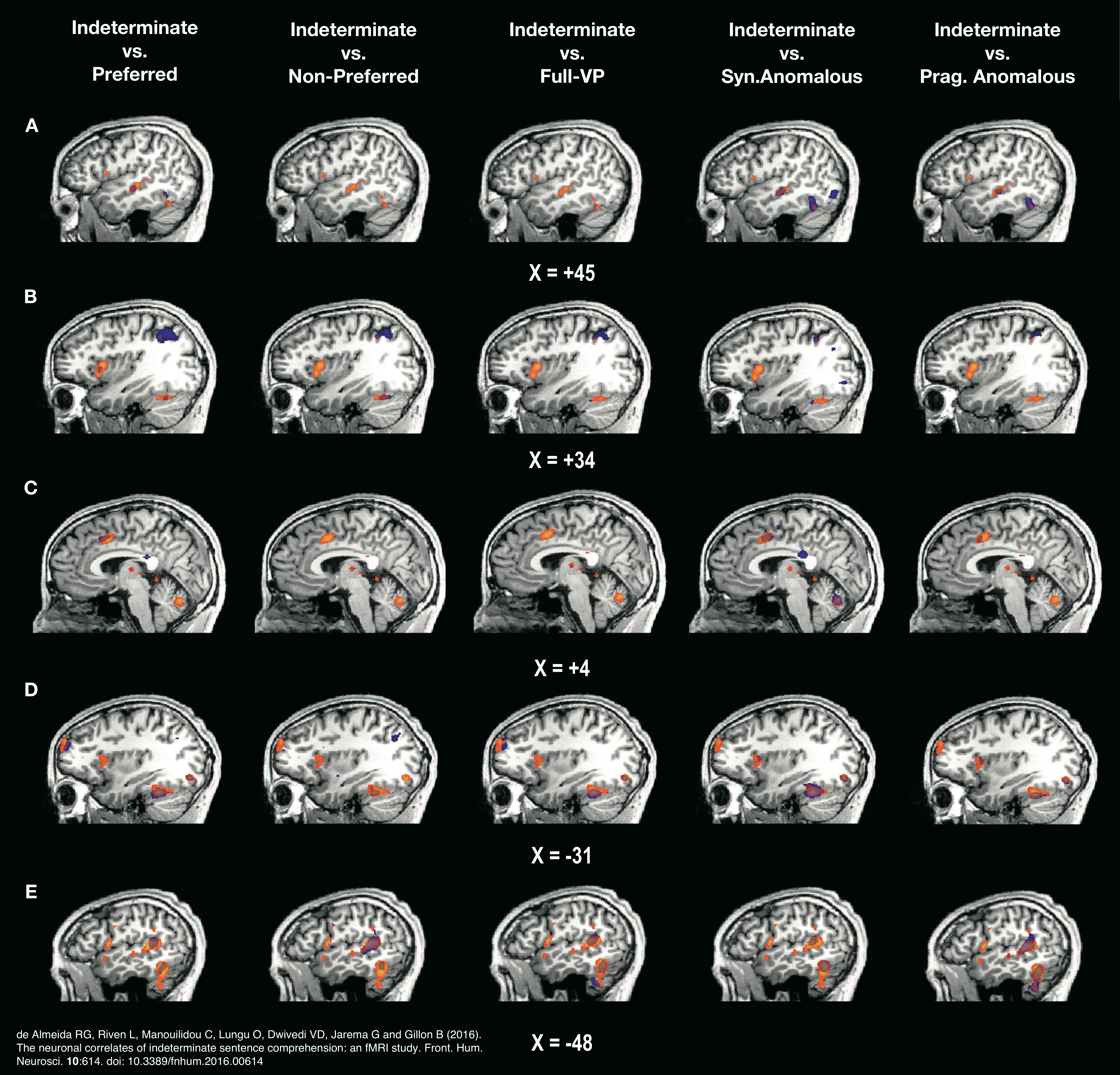

Supplement: Supplementary file 1 [file Image_3.tiff]
